# Factors affecting prognosis in traumatic cerebral contusions: A protocol for a systematic review and meta-analysis

**DOI:** 10.1371/journal.pone.0319146

**Published:** 2025-02-25

**Authors:** Chao Li, Zhaoyin Su, Shulu Deng, Binhao Zhang, Junlong Qin, Kun Wu, Yanzong Zhao, Yao Liu

**Affiliations:** 1 Department of Emergency, The Brain Hospital of Guangxi Zhuang Autonomous Region, Liuzhou, China; 2 The First School of Clinical Medicine, Lanzhou University, Lanzhou, China; 3 Department of Cardiovascular Surgery, Central South Hospital of Wuhan University, Wuhan, China; 4 Department of Orthopedic Surgery, The Seventh Affiliated Hospital of Southern Medical University, Foshan, China; 5 School of Stomatology, Lanzhou University, Lanzhou, China; National center for chronic and non-communicable diesease prevention and control, CHINA

## Abstract

**Background:**

Traumatic cerebral contusion (CC) is a severe type of injury among traumatic brain injury (TBI) patients. Individuals with traumatic CC typically exhibit rapid deterioration in their condition, leading to increased mortality rates. Despite this, there is a gap in evidence-based research. This study aims to identify the risk factors associated with adverse outcomes in patients with traumatic CC, with a particular focus on relevant biomarkers. Mortality will be the primary outcome, while the Glasgow Coma Scale (GCS) score will be considered as a secondary outcome.

**Methods and analysis:**

We intend to conduct a comprehensive search through multiple Chinese and English repositories, covering the duration from the establishment of these databases up to the current era, in order to pinpoint appropriate studies. Additionally, a manual search of the references within the included literature and other pertinent works will be undertaken. The primary endpoint of this study will be the survival status of patients with traumatic brain contusion. Meta-analysis will be executed using STATA 16.0 (Stata Corporation, College Station, TX). Article selection and data extraction will be performed independently by two reviewers. The assessment of bias risks will be conducted via the Cochrane Collaboration’s tool. Depending on the heterogeneity evaluation, either a fixed-effect model or a random-effects model will be applied. Subgroup and sensitivity analyses will be conducted as needed. The examination of publication bias will be carried out, and the quality of evidence for the primary outcomes will be graded.

**Trial registration number:** CRD42023389456

## 1. Introduction

Traumatic brain injury (TBI) is a leading cause of death and disability worldwide, second only to limb fractures, and is often referred to as a “silent epidemic” [1]. In China and similar developing countries, the prevalence of severe TBI is approximately 13 per 100,000 individuals, with a mortality rate ranging from 30% to 40%, and disabilities affecting up to 60% of survivors [2]. Despite improvements in the availability of advanced diagnostic tools and treatment options, TBI remains a major public health concern.

In line with trends observed in many Western nations, the mortality rate for severe TBI in China has gradually decreased in recent years [3]. However, with mortality still exceeding 30% and adverse outcomes affecting more than 60% of severe cases, the burden of TBI remains significant [4]. One of the most severe forms of TBI is cerebral contusion (CC), which occurs in 20–30% of TBI cases [5]. CC is characterized by acute traumatic intracerebral hemorrhage (TICH) and is associated with high rates of mortality and disability.

The progression of TICH refers to an increase in hemorrhagic elements within the contusion, as seen in post-imaging follow-up. Approximately 38–59% of CC cases experience TICH expansion, particularly within the first few hours following the trauma [6]. While most enlarging TICHs do not require surgical intervention, some patients may develop worsening neurological function, mass effect, intractable cerebral edema, increased intracranial pressure, or even fatal brain herniation. These complications impose substantial burdens on patients, families, and healthcare systems [7].

Studies have shown that up to 20% of TBI patients require surgery due to TICH expansion, emphasizing the critical need for a deeper understanding of the early factors influencing CC progression. Early detection of TICH enlargement and close monitoring of patients’ conditions are essential for improving outcomes and for the judicious allocation of medical resources [8]. However, current research on predictive risk factors for brain hemorrhage has primarily focused on spontaneous hemorrhage, with limited attention given to factors influencing the progression of traumatic CC. This gap in knowledge has led to inconsistent treatment approaches and suboptimal patient care.

In response, our study aims to conduct a meta-analysis to examine the risk factors affecting the condition and prognosis of traumatic brain contusions, with a particular focus on identifying factors that significantly influence patient outcomes. Specifically, we will explore the role of relevant biochemical markers in predicting mortality in patients with traumatic CC, with the Glasgow Coma Scale (GCS) score used as a secondary outcome measure. Our findings will provide a theoretical framework for improving the treatment of patients with traumatic brain contusions. To the best of our knowledge, this will be the first systematic review on this topic. Therefore, we present this protocol for the meta-analysis and systematic review.

## 2. Materials and methods

### 2.1. Protocol and registration

This paper presents a protocol for a systematic review and meta-analysis. The synthesis follows the guidelines outlined in the Preferred Reporting Items for Systematic Review and Meta-Analysis Protocols (PRISMA-P) 2015 and is registered with PROSPERO under registration number CRD42023464826. The review will be based on existing scholarly literature and does not require ethical approval. The results will be presented in subsequent publications.

### 2.2. Inclusion criteria

The selection criteria for this research follow the PICOS structure (population, intervention/exposure, comparator, outcomes, study design) [9]. Studies fulfilling these criteria will be encompassed, with no limitations pertaining to language or the year of publication.

#### Population.

The study will encompass adult individuals (18 years and older) diagnosed with traumatic CC, without any exclusions based on nationality, race, ethnic background, age, gender, or professional status.

#### Exposure.

The variables identified in the studies that may influence the condition and prognostic outcomes of individuals with traumatic CC will be considered, with a particular focus on biochemical indicators.

#### Comparator.

Individuals who are not subjected to the identified exposure factors will serve as the comparison group.

#### Outcomes and Assessments.

**Primary Outcome:** The mortality rate among patients suffering from traumatic CC.

**Secondary Outcomes:** GCS score.

**Study Designs:** Eligible for inclusion are randomized controlled trials, non-randomized controlled studies, as well as observational research designs.

### 2.3. Exclusion criteria

Investigations focusing on populations affected by ischemic stroke, spontaneous intracerebral hemorrhage, chronic subdural hematoma, or other types of non-traumatic cerebral hemorrhages will be excluded.

Research papers that lack comprehensive clinical data, even after diligent efforts to reach out to the respective authors, will not be included.

Exclusion will apply to correspondence, conference summaries, editorial pieces, case studies, review articles, and any studies lacking clinical data.

Accessible full-text scholarly works that cannot be located through thorough search efforts will not be considered for inclusion.

### 2.4. Study selection and search strategy

An extensive literature search will be performed across multiple databases, including PubMed, Embase, Web of Science, The Cochrane Library, CNKI (China National Knowledge Infrastructure), VIP (WeiPu), SinoMed, and Wanfang, covering all available records from the inception of each database to the current date. The search strategy has been carefully designed with input from a professional librarian and information specialist to ensure comprehensiveness and precision. It is informed by existing literature and incorporates Medical Subject Headings (MeSH) terms, keywords, and synonyms relevant to the research topic.

Search terms will include variations of “traumatic brain injury,” such as “traumatic cerebral contusions,” “TBI,” “head injury,” “cerebral blunt trauma,” and “diffuse axonal injury,” combined with terms like “adverse prognosis,” “unfavorable outcome,” “risk factors,” “predictors,” “impact factors,” “determinants,” and “predictive indicators.” To maximize retrieval, Boolean operators (AND/OR) and database-specific controlled vocabulary will be applied. For instance, MeSH terms will be used in PubMed, and EMTREE terms in Embase, supplemented by title and abstract keywords tailored to the syntax of each database.

To ensure thoroughness, the reference lists of included studies will be screened for additional articles, and gray literature will be identified via OpenGrey. Additionally, clinical trial registries, including ClinicalTrials.gov and the WHO International Clinical Trials Registry Platform (ICTRP), will be searched to identify ongoing or unpublished randomized controlled trials (RCTs). Authors of ongoing studies will be contacted directly to gather the most recent findings. These steps aim to reduce the risk of publication bias and to capture a comprehensive scope of relevant research.

### 2.5. Search terms for PubMed

#1: ((traumatic brain injury[MeSH Terms]) OR (traumatic brain injuries[Title/Abstract])) OR (TBI[Title/Abstract])) OR (brain injuries, traumatic(Title/Abstract])) OR (head injury[Title/Abstract])) OR (cerebral contusion(Title/Abstract])) OR (brain contusion[Title/Abstract])) OR (closed head injury[Title/Abstract]) OR (diffuse axonal injury[Title/Abstract]))

#2: ((((prognosis[MeSH Terms]) OR (outcome[Title/Abstract])) OR (mortality[Title/Abstract])) OR (recovery[Title/Abstract])) OR (functional outcome[Title/Abstract])) OR (neurological recovery[Title/Abstract])) OR (cognitive outcome[Title/Abstract])) OR (disability[Title/Abstract])) OR (quality of life[Title/Abstract])

#3: ((((risk factors[MeSH Terms]) OR (predictors[Title/Abstract])) OR (determinants[Title/Abstract])) OR (factors[Title/Abstract])) OR (clinical predictors[Title/Abstract])) OR (biomarkers[Title/Abstract])) OR (severity score[Title/Abstract])

#4: #1 AND #2 AND #3

### 2.6. Literatures collection and organization

**Fig 1:**
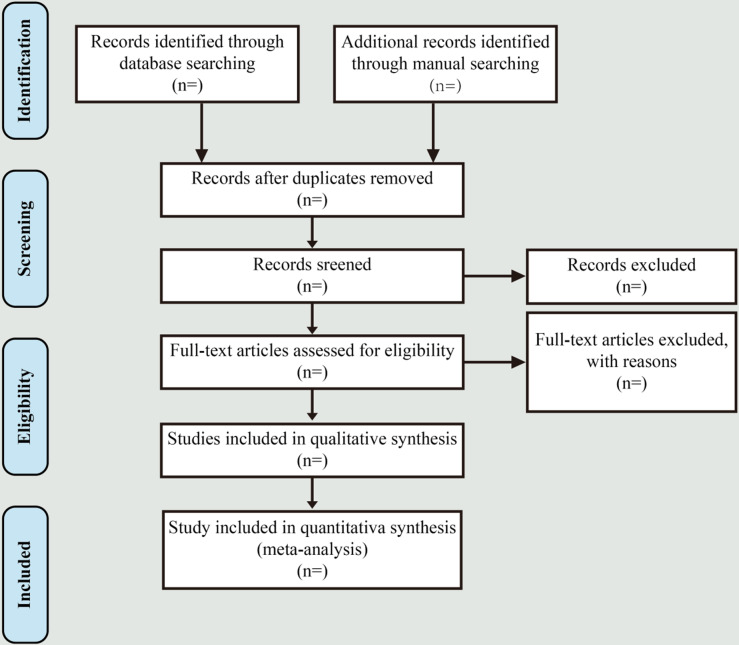
Flow chart of literatures screening.

All identified literature will be imported into the NoteExpress reference management tool. After duplicates are removed, Zhaoyin Su and Yao Liu, as the first and second reviewers, will conduct article selection based on the previously established criteria. The process will begin with an initial screening of titles and abstracts to assess relevance. Subsequently, both reviewers will independently examine the full texts of all potentially relevant studies. The article selection process is illustrated in Fig 1. In case of discrepancies between the two reviewers, a third expert will be consulted to reach a consensus.

### 2.7. Data extraction and management

Two other independent reviewers will employ preformatted Excel templates to extract a set of predetermined data. The extracted information will include study characteristics (such as the primary authors, journal, publication year, study design, blinding methods, and inclusion criteria), participant characteristics (e.g., diagnostic criteria for traumatic CC, age range, and gender ratio), exposure factors (e.g., biochemical marker values), outcome measures (e.g., mortality and GCS scores), as well as follow-up duration. Where quantitative data are not directly provided in the text or additional materials, Engauge Digitizer 11.1 will be used to extract data from graphical representations. For studies with incomplete data, the reviewers will reach out to the corresponding author via email to request the missing information, offering to acknowledge their assistance. If no response is received within two weeks of the initial inquiry, further attempts to contact the study’s researcher will be halted, and the study will be excluded from this systematic review and meta-analysis. Effect estimates for the outcomes of interest will be extracted for statistical analysis, with a preference for adjusted data that controls for confounding variables. To reduce bias and errors in the data extraction process, two researchers will independently extract data from the selected literature. Following extraction, a cross-verification process will take place, with any discrepancies resolved through discussion. If an agreement cannot be reached, a third reviewer will be involved to facilitate a resolution.

### 2.8. Data synthesis and statistical analysis

Statistical analyses and graphical representations will be executed utilizing STATA 16.0. One researcher will be responsible for entering the post-treatment mean values and standard deviations into the software, while a second researcher will cross-check these entries for correctness. The assessment of data heterogeneity will be performed via the chi-square and I^2 tests. In the event of non-significant heterogeneity (P ≥  0.10 or I^2 ≤  50%), the fixed-effect model will be chosen for the meta-analysis. On the other hand, if significant heterogeneity is detected (I^2 > 50% or P < 0.10), the random-effects model will be applied. To examine the impact of baseline attributes on the variability of primary outcomes, subgroup analyses will be carried out focusing on the following patient-related factors: (1) Glasgow Coma Scale scores of the patients, and (2) cerebral perfusion pressure indices. Additionally, subgroup analyses will be extended to different study design categories, complemented by meta-regression analyses, to explore potential origins of heterogeneity. We will strive to precisely identify and address heterogeneity exceeding 50%. In cases where heterogeneity is excessively high, a qualitative synthesis will be conducted. To ascertain the stability of our findings, a sensitivity analysis will be performed, removing studies one at a time to assess the impact on the overall results. A significance level of p < 0.05 will be denoted as statistically significant. Following this analytical process, a detailed examination and comparison of the outcomes will be undertaken to gauge the consistency of the study results. This rigorous evaluation is intended to confirm the dependability and veracity of our findings.

### 2.9. Assessment of risk of bias

A duo of investigators will independently apply the Cochrane Collaboration’s Risk of Bias tool [10] to gauge the potential bias in the randomized controlled trials (RCTs) incorporated into this review. The categorization of studies into high, low, or unclear risk of bias will be determined by examining various domains of bias, including: 1) selection bias, encompassing random sequence generation and allocation concealment; 2) performance bias, concerning the blinding of participants and outcome evaluators; 3) attrition bias, associated with incomplete outcome data; 4) reporting bias, pertaining to the selective reporting of results; and 5) other conceivable sources of bias. For non-randomized controlled trials, the methodological quality will be evaluated using the Newcastle-Ottawa Scale [11], which consists of three main domains: selection of participants, comparability of study groups, and assessment of outcomes. Each study will be assigned a score out of a possible nine stars, with scores ranging from 0 to 5 stars indicating low quality and 6 to 9 stars indicating high quality. Any conflicts in the evaluation process will be resolved through dialogue between the two assessors or, if required, by seeking the input of a third reviewer. The outcomes of these assessments will be compiled into a summary table. In cases where bias is deemed substantial, we may need to reconsider the literature gathered or potentially replace certain studies to enhance the synthesis of data.

The examination of reporting bias will be undertaken as necessary to corroborate the validity of the study findings. Should the review include more than ten studies, the assessment of reporting biases will involve the use of Egger’s test and a visual examination of the funnel plot, which plots the estimated effect size (on the horizontal axis) against its standard error (on the vertical axis). An asymmetrical funnel plot, particularly an inverted one, may suggest the presence of publication bias.

## 3. Discussion

Traumatic brain injury (TBI) presents a formidable challenge, affecting individuals profoundly and imposing significant burdens on the socioeconomic landscape. In the United States alone, an estimated 1.4 million incidents of TBI occur each year, with a staggering 90,000 individuals left to navigate long-term disabilities as a result [12]. China, housing approximately 18% of the global population, also contends with a substantial caseload of TBI patients, surpassing most nations in absolute numbers, where over 60% of cases lead to disabling outcomes [13]. The course of traumatic hemorrhage is but the starting point of a complex biochemical cascade, exacerbating neuronal damage and clinical deterioration. In particular, secondary injury prolongs hospital stays, escalates morbidity, and raises mortality rates. As of now, no consensus therapeutic intervention exists to halt the progression of lesions [14]. Among the most devastating forms of TBI is hemorrhagic cerebral contusion, inflicting irreversible harm upon brain tissue [15]. The gravity of the injury is linked not only to the initial impact but also to the subsequent secondary responses triggered by external forces, such as cerebral edema (CE), elevated intracranial pressure (ICP), microvascular dysfunction, epileptic seizures, and more [16]. Imaging and laboratory results from patients reveal numerous prognostic indicators, vital for timely and precise diagnosis. However, the body of evidence-based medicine lacks a comprehensive synthesis of risk factors for poor outcomes in those with traumatic cerebral contusions, complicating the development of clinical guidelines. Our systematic review seeks to provide a stronger evidentiary base for the risk factors influencing negative prognoses in these patients, thereby drawing greater focus to this critical field of research. Our study is not without its limitations; the quality of the primary research will undoubtedly influence our conclusions, and variability in condition severity and treatment approaches may introduce substantial heterogeneity. If sufficient data permits, subgroup analyses will be pursued. In essence, we endeavor to conduct a groundbreaking systematic review and meta-analysis to evaluate the determinants of adverse outcomes in patients with traumatic cerebral contusions, with the goal of enhancing diagnostic and therapeutic strategies for those suffering from this condition.

## 4. Strengthens and limitations

(1) This scholarly endeavor constitutes the pioneering exploration aimed at systematically appraising the risk factors associated with adverse clinical outcomes in patients with traumatic cerebral contusions.
(2) An extensive literature search will be executed across a array of databases, encompassing both Chinese and English linguistic resources.
(3) The procurement and synthesis of data will rigidly conform to the methodological standards delineated in the Preferred Reporting Items for Systematic Reviews and Meta-Analyses (PRISMA) checklist and the Cochrane Handbook for Systematic Reviews of Interventions.
(4) Profound subgroup analyses are contemplated, wherein data will be segmented based on therapeutic interventions and the gradation of pathological severity.
(5) The disparate quality of the original investigations may engender significant heterogeneity, thereby influencing the robustness of the empirical findings.
